# Stationäre chirurgische Versorgung in Großschadenslagen und Katastrophen – Grundlagen, Ziele, Konzepte, Vorbereitung

**DOI:** 10.1007/s00104-023-01976-w

**Published:** 2023-11-09

**Authors:** Axel Franke, Wolfgang Lehmann, Thomas Wurmb

**Affiliations:** 1 Sektion Unfallchirurgie, Klinik für Unfallchirurgie, Orthopädie, Hand- und Rekonstruktive Chirurgie, Verbrennungsmedizin, BundeswehrZentralkrankenhaus Koblenz, Rübenacher Straße 170, 56072 Koblenz, Deutschland; 2 Klinik für Unfallchirurgie, Orthopädie und Plastische Chirurgie, Universitätsmedizin Göttingen, Robert-Koch-Straße 40, 37099 Göttingen, Deutschland; 3 Klinik und Poliklinik für Anästhesiologie, Intensivmedizin, Notfallmedizin und Schmerztherapie, Sektion Notfall- und Katastrophenmedizin, Universitätsklinikum Würzburg, Würzburg, Deutschland

**Keywords:** Gesundheitssystem, Katastrophenmedizinische Patientenversorgung, Kompensierte Krisenversorgung, Behandlungskapazität, Zentrale Führungsstruktur, Notfallversorgung, Healthcare system, Patient medical treatment in catastrophes, Compensated crisis care, Treatment capacity, Central management structure, Contingency care

## Abstract

**Hintergrund:**

Der Krieg in der Ukraine und die SARS-CoV-2(„severe acute respiratory syndrome coronavirus type 2“)-Pandemie haben die Resilienz unseres Gesundheitssystems in den Fokus einer breiten Diskussion gerückt. Die Vorbereitung auf solche Schadenslagen steht im Zeichen des Verhältnisses aus verfügbarer Behandlungskapazität und einen weit über die Norm hinausgehenden Bedarf. Ziel eines resilienten Gesundheitssystems muss es sein, auf solche Ausnahmesituationen adäquat zu reagieren. Insbesondere in akuten Katastrophen- und Großschadenslagen gilt es, möglichst lange medizinische Standards und eine individualmedizinische chirurgische Versorgung aufrechtzuerhalten.

**Material, Methode und Ziel der Arbeit:**

Zielsetzung der vorliegenden Arbeit ist es, anhand definierter Schadenslagen die aktuellen Begrifflichkeiten zur katastrophenmedizinischen Patientenversorgung aus chirurgischer Perspektive zu erläutern, vorhandene weiterzuentwickeln und mögliche Konzepte der Krisenversorgung anhand dreier schematisch dargestellter Szenarien darzustellen. Darüber hinaus werden allgemeine Reaktionsmöglichkeiten zur Mobilisation von Behandlungskapazitäten beschrieben.

**Ergebnisse:**

Um die medizinische Versorgungsqualität in einer Schadenslage einheitlich zu erfassen, ist es sinnvoll, von den Stadien der Individualstandardversorgung, kompensierter Krisenversorgung und dekompensierter Krisenversorgung zu sprechen. Im Rahmen einer Großschadenslage oder einer Katastrophe werden traumatologisch-chirurgische Patienten überwiegen und es muss das Ziel sein, das Stadium einer kompensierten Krisenversorgung zu erhalten oder wieder herzustellen. Je nach Ausdehnung der Schadenslage kann dies nur unabhängig von den Landesgrenzen und durch eine übergeordnete zentrale Führungsstruktur zeitnah realisiert werden. Für eine flächendeckende Bereitstellung chirurgischer Behandlungskapazitäten ist die Darstellung eines kontinuierlichen Lagebildes mit aktuellen Ressourcen und Strukturdaten der Krankenhäuser in der betroffenen Region erforderlich.

**Schlussfolgerung:**

Ziel aller Bemühungen und Vorbereitungen muss es sein, die Krankenhäuser dauerhaft zu ertüchtigen und bezüglich der katastrophenmedizinischen Bewältigung einer Schadenslage auszubilden und zu entwickeln. Es ist dabei wichtig, einen allgemeinen Konsens über Begrifflichkeiten, Art der Versorgung und die taktisch strategischen Grundsätze der chirurgischen Versorgung zur Bewältigung einer Katastrophe oder Schadenslage zu etablieren.

## Abstract (en)

**Background:**

The war in Ukraine and the severe acute respiratory syndrome coronavirus type 2 (SARS-CoV-2) pandemic have moved the resilience of the healthcare system in Germany into the focus of a broad discussion. The preparation for such a damage situation is characterized by the relationship between the available treatment capacity and needs which go far beyond the norm. The aim of a resilient healthcare system must be to adequately react to such exceptional situations. Particularly in acute disaster and mass casualty situations, medical standards and an individualized surgical treatment must be maintained for as long as possible.

**Material, method and objective:**

The aim of this article is to elucidate the current terminology on medical treatment of patients in disasters from a surgical perspective, to further develop available concepts and possible concepts of crisis management based on three schematically presented scenarios. Furthermore, the general reaction possibilities for mobilization of treatment capacities are described.

**Results:**

In order to uniformly collate the quality of medical treatment in a damage situation, it is meaningful to include the stages of individualized treatment, compensated crisis care and decompensated crisis care. Within the framework of a mass casualty situation or a disaster, traumatological and surgical patients are predominant and the aim must be to maintain or restore the stage of a compensated crisis management. Depending on the extent of the damage situation, this can only be realized in a timely manner independent of state boundaries and by a superordinate central management structure. For a comprehensive provision of surgical treatment capacities, the depiction of a continuous overview of the situation with current resources and structural data of the hospitals in the affected region is necessary.

**Conclusion:**

The aim of all efforts and preparations must therefore be to durably strengthen hospitals and to train and develop this with respect to coping with a damage situation in disaster medicine. In this respect it is important to establish a consensus on terminology, the type of treatment and the tactical strategic principles of surgical treatment to cope with a disaster or damage situation.

## Hintergrund und Ziel der Arbeit

Nicht zuletzt durch den Krieg in der Ukraine und die SARS-CoV-2(„severe acute respiratory syndrome coronavirus type 2“)-Pandemie ist die Resilienz der weltweiten Gesundheitssysteme und die Robustheit der Krankenhausstrukturen für eine notfall- und katastrophenmedizinische Krisenversorgung in den Fokus aktueller Diskussionen gerückt. In diesem Zusammenhang spielt die Vorbereitung auf Katastrophen und Großschadenslagen eine herausragende Rolle.

Katastrophen und Großschadenslagen werden, insbesondere bei unerwartetem Eintreten, mit massiver Schädigung von Menschen und Infrastruktur einhergehen und zu einer akuten Überlastung der medizinischen Versorgungsstrukturen führen. Hierbei führt die direkte Schädigung des Einzelnen in der Mehrzahl der Fälle zum Vorliegen chirurgisch-traumatologischer Krankheitsbilder, wohingegen der Ausfall ambulanter und stationärer Behandlungseinrichtungen die Überlastung noch funktionsfähiger Versorgungseinrichtungen mit Krankheitsbildern verursacht, die der Gesamtmorbidität der Gesellschaft entspricht [1, 2].

Ziel der Vorbereitung auf solche Schadenslagen muss es sein, in einer akuten, aber auch lang andauernden Belastungssituation die anerkannten und medizinischen Standards aufrechtzuerhalten, um eine möglichst individualmedizinische chirurgische Versorgung sicher zu stellen. Neben einer gut strukturierten Führung, einer kontinuierlichen Lagedarstellung und Lagebewertung sind das Verhältnis von Behandlungskapazität und Behandlungsbedarf hierbei die entscheidenden Parameter [3, 20].

Für ein allgemeines Verständnis im Rahmen der Vorbereitung und Lagebewältigung ist ein möglichst gemeinsamer Sprachgebrauch von entscheidender Bedeutung.

Ziel dieser Arbeit ist es daher, anhand erwartbarer Ereignisse aktuelle Begrifflichkeiten zu beschreiben, vorhandene weiterzuentwickeln und mögliche Konzepte der Krisenversorgung darzustellen.

Darüber hinaus werden Reaktionsmöglichkeiten zur Mobilisation von Behandlungskapazitäten erläutert. Diese orientierenden Überlegungen werden in einer zweiten Arbeit mit vorhandenen Strukturen der Traumanetzwerkkliniken und bekannten Versorgungskapazitäten in ein Verhältnis gestellt [9].

Diese Ausarbeitung ist erforderlich, da beispielsweise medizinische Stellungnahmen in der Literatur bezüglich der Situation in Deutschland mehr als 10 Jahre alt sind. Darüber hinaus werden bei den aktuellen Entwicklungen im Gesundheitssystem und der Etablierung und Zertifizierung der Kliniken in den Traumanetzwerken der Deutschen Gesellschaft für Unfallchirurgie (DGU) die neuen Begrifflichkeiten der Krisenversorgung nicht berücksichtigt [1, 2, 17].

## Erwartbare Schadensereignisse und die daraus resultierenden Belastungen für das (stationäre) Gesundheitssystem

### Szenarien

**Figure 1:**
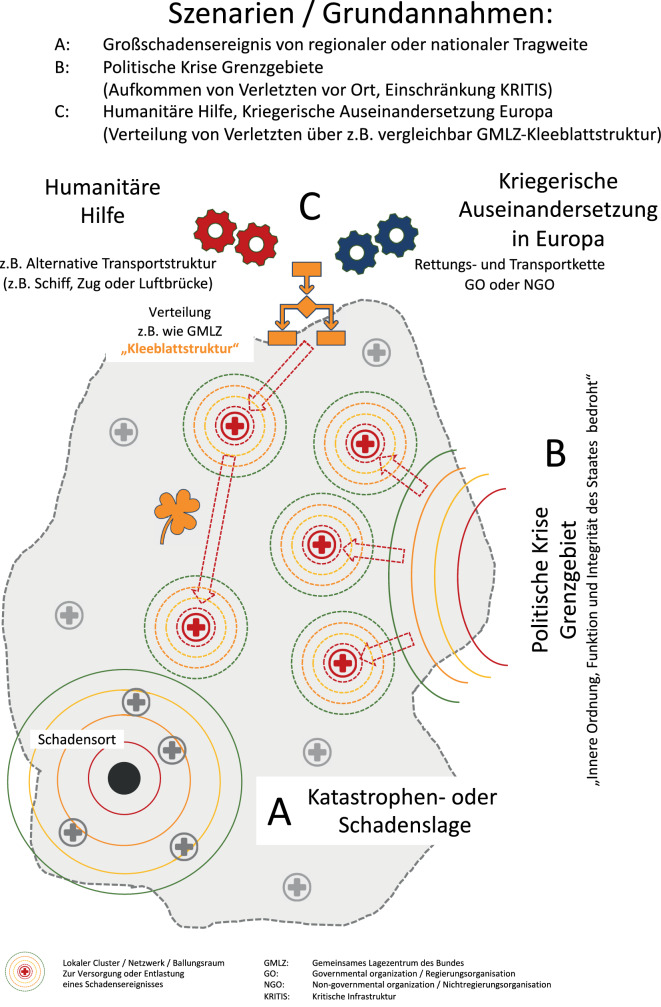

Ein Gesundheitssystem kann grundsätzlich durch drei verschiedene Szenarien im Sinne einer Katastrophe oder Großschadenslage akut belastet werden. Die hier gewählte Darstellung der Szenarien dient einer schematischen Strukturierung. Selbstverständlich kann es im Ereignisfall zu entsprechenden Mischkonstellationen kommen (Abb. 1).

#### Szenario A.

Durch ein (Groß‑)Schadensereignis (z. B. Busunfall, Zugunglück, Flugzeugabsturz in einer bevölkerungsreichen Region, Überflutung, Erdbeben, Industrieunfall) kommt es zu einem kurz-, mittel- oder längerfristigen lokalen oder regionalen Aufkommen von Verletzten, Erkrankten und Betroffenen (z. B. Flutkatastrophe im Ahrtal oder Terroranschlag 2001 in New York). Hier determiniert die Art des Ereignisses, die Dynamik der Entstehung und die Einbeziehung kritischer Infrastruktur, die Art der Verletzungen, die Anzahl der Patienten und die voraussichtliche Dauer der Belastung. Die Ausdehnung der katastrophenmedizinischen Krisenversorgung lagert sich konzentrisch um den Schadensort.

#### Szenario B.

Im Rahmen eines flächigen und lang andauernden Schadensereignisses (z. B. bewaffneter Konflikt, Überflutung, Erdbeben) kann es zu einem dynamischen Aufkommen einer großen Anzahl von Verletzten kommen. Einschränkungen der kritischen Infrastruktur einer ganzen Nation werden in einem solchen Szenario die medizinische Versorgung weiter erschweren. Flüchtlingsbewegungen und die Versorgung der Bevölkerung sind weitere systembelastende Faktoren. Ordnung, Funktion und Integrität eines Staates werden von außen bedroht (z. B. Krieg).

#### Szenario C.

Durch eine militärische Auseinandersetzung, die weit über das Gebiet eines einzelnen Staates hinausgeht oder aber im Rahmen der humanitären Hilfe für andere Staaten, erfolgt die Verteilung von Erkrankten oder Verletzten aus der betroffenen Region. Die von außerhalb des eigenen Staatsgebiets über einen koordinierenden Verteilungsmechanismus (z. B. Gemeinsame Melde- und Lagezentrum des Bundes [GMLZ] oder Kleeblattsystem) Transportierten belasten in Abhängigkeit von Anzahl und Art der aufkommenden Verletzten die lokalen oder regionalen Versorgungsstrukturen, die sich an der medizinischen Bewältigung in dieser Situation beteiligen.

Unabhängig von den drei dargestellten exemplarischen Szenarien ist grundsätzlich die zusätzliche Zerstörung und Funktionseinschränkung kritischer Infrastruktur durch Terrorattacken, hybride Bedrohung und Cyberangriffe oder aber der Einsatz von Raketen oder Drohnen denkbar. Dies kann lokal, regional und national zu einer Reduktion der Behandlungskapazitäten führen ohne ggf. mit einem Aufkommen von Verletzen und Erkrankten einherzugehen. Zur Vereinfachung bezieht sich dieser Artikel auf die Szenarien A, B und C.

### Begrifflichkeiten und Darstellung der Versorgungssituationen am Beispiel des Szenarios A

Die Fähigkeit zur medizinischen Versorgung im Rahmen eines Schadensereignisses durch Krankenhäuser wird durch deren Funktionalität und mobilisierbare Behandlungskapazität bestimmt. Hier sind 4 Konstellationen denkbar [12, 18].

1. Die Behandlungskapazität (K) ist größer als der aktuelle Bedarf (B; K > B: Status Grün, *Standardversorgung*).
2. Die Behandlungskapazität entspricht dem aktuellen Bedarf und das Ereignis ist abgeschlossen (K = B: Status Gelb, *kompensierte Krisenversorgung*).
3. Die Behandlungskapazität entspricht gerade noch dem aktuellen Bedarf, das Ereignis ist nicht abgeschlossen und der Bedarf steigt weiter an (K = B: Status Orange, *gefährdete kompensierte Krisenversorgung*).
4. Trotz Einleiten und Umsetzen aller verfügbaren Gegenmaßnahmen ist die Behandlungskapazität kleiner als der aktuelle Bedarf (K < B: Status Rot, *dekompensierte Krisenversorgung*).

Ziel bei allen Planungen und Vorbereitungen auf solche Schadensereignisse muss es sein, das System im Bereich der Standardversorgung oder der kompensierten Krisenversorgung zu halten.

Zu den hierfür notwendigen Vorbereitungen zählen ein funktionsfähiger Krankenhausalarm- und -einsatzplan, die Möglichkeit zur Personalrekrutierung, Verfahrensanweisungen und das Schulen einer prioritätenorientierten taktischen Versorgung, ausreichende Materialressourcen ggf. eingelagerte Reserven, Führungs- und Lagemeldungsstrukturen sowie die Möglichkeit einer strategischen und akuten Patientenverlegung.

Zu einer *dekompensierten Krisenversorgung* kommt es vor allem dann, wenn Vorbereitungen gar nicht oder nicht ausreichend getroffen werden, die Behandlungskapazität schnell erschöpft ist, die akuten Fähigkeiten zur Steigerung der Behandlungskapazität nicht gegeben sind oder die Schadenslage das erwartbare Maß überschreitet. Zu diesem Zeitpunkt zielen alle Maßnahmen auf das schnelle Wiedererreichen einer *kompensierten Krisenversorgung*.

In Abhängigkeit vom Szenario ist es möglich, dass unmittelbar am Schadensort durch die Schädigung kritischer Infrastruktur gegebenenfalls keine medizinische Versorgung in funktionsfähigen Krankenhausstrukturen mehr möglich ist. Auch dies muss in den Planungen bedacht werden. In einem solchen Fall erfolgt das Retten der Verletzten und der Weitertransport in unmittelbar benachbarte funktionsfähige Krankenhäuser.

Wenn es trotz aller Anstrengungen und einer optimalen Vorbereitung nicht gelingt, die Behandlungskapazität an den Bedarf anzugleichen, bleibt die dringende Notwendigkeit zur Patientenversorgung dennoch bestehen. In einer solch katastrophalen Situation ist es möglich, dass normative Vorgaben bezüglich z. B. Hygiene, Arbeitszeitgesetz, individualmedizinischer Behandlungsstandards vorübergehend nicht sichergestellt oder aufrechterhalten werden können.

Zur Entlastung der unmittelbar betroffenen Krankenhäuser können Patienten, die lokal nicht entsprechend den Standards behandelt werden können, in andere Krankenhäuser verlegt werden. Dies führt zu einer Belastung benachbarter Krankenhausstrukturen. Auch hier kommt es nun auf die Fähigkeit des Systems an, die Behandlungskapazität im erforderlichen Umfang an den Bedarf anzupassen. Hier kommt es nun ebenfalls zu einer akuten Kapazitätsüberschreitung, was zur Folge haben kann, dass die Einhaltung medizinischer Standards auch hier gefährdet ist. Es kommt zu einer noch kompensierten aber *gefährdeten Krisenversorgung* [19, 20]. Diese zeichnet sich dadurch aus, dass zwar nicht alle Standards der Arbeits- und Prozessorganisation regelhaft abgebildet werden können, eine Behandlung aber noch unter weitestgehender Einhaltung der medizinischen Standards erfolgen kann. Hier erfolgt teilweise schon die Behandlung unter der Maßgabe, ein „Überleben mit möglichst bestem funktionellem Outcome“ für alle zu ermöglichen.

## Reaktionsmöglichkeiten zur Aufrechterhaltung oder Wiederherstellung einer kompensierten Krisenversorgung

### Allgemeine kurzfristige Maßnahmen

Führung, Raumordnung, Personalkonzepte, Materialvorräte und Sichtung sind wesentliche Werkzeuge zur akuten Steigerung oder Fokussierung der Behandlungskapazität. In der akuten Mangelsituation müssen ggf. vorübergehend die Behandlungsziele einer individualmedizinischen Standardversorgung verlassen werden (s. oben). Dies geschieht, um zunächst für allen Patienten die bestmöglichen Voraussetzungen für ein Überleben sicherzustellen.

Eine Grundlage hierfür ist die Eingangssichtung am Krankenhaus (Ex-ante-Triage), um die durch das auslösende Schadensereignis hervorgerufenen unmittelbaren (Sichtungskategorie [SK] I rot) oder mittelbaren (SK II gelb) lebensbedrohlichen Folgezustände zu erkennen. Die Eingangssichtung ist in einer solchen Lage die individuelle Kategorisierung jedes einzelnen Patienten und dient dem Erkennen einer potenziell lebensbedrohlichen Situation [6–8].

Die Eingangssichtung (*Kategorisierung*) am aufnehmenden Krankenhaus ist die Voraussetzung für eine *Priorisierung *(Art, Ressourcenansatz und Reihung) und *Disposition* der weiteren Maßnahmen zur *Realisierung* der notfallmedizinischen und -chirurgischen Behandlung [5].

Im Rahmen der Sichtung werden Patienten identifiziert, die unmittelbare (SK I rot) oder mittelbare (SK II gelb) lebensbedrohliche Folgezustände nach der initialen Verletzung und Erkrankung aufweisen, und mit der Priorisierung auf die Sicherstellung des Überlebens aller Patienten erfolgt die notfallmedizinische Stabilisierung nach etablierten Algorithmen (z. B. Primary Survey ATLS [Advanced Trauma Life Support], TDSC [Terror and Disaster Surgical Care] AUC [Akademie der Unfallchirurgie]; [8]).

Nach der Sichtung und der initialen Stabilisierung ist die weitere Notfallbehandlung zu priorisieren. In dieser Situation ist die weitere Planung und Reihung der Behandlung nur in Kenntnis der vorhandenen Behandlungsressourcen (z. B. Operationskapazität, Blutprodukte) möglich.

Im eingangs beschriebenen Szenario A muss die Behandlungskapazität akut gesteigert werden. Hierzu kann es erforderlich werden, z. B. die Durchführung elektiver Eingriffe zwischenzeitig auszusetzen, um Behandlungskapazitäten für die Notfallbehandlung zu generieren [21].

Operative Eingriffe, die gegebenenfalls mit einer Verschlechterung einer vorhandenen (z. B. malignen) Grunderkrankung einhergehen, konkurrieren in dieser Situation mit den erforderlichen Notfalleingriffen der von außen zugewiesenen Patienten und müssen individuell bewertet werden. Auch hier könnten Patienten in unbeteiligte Regionen verlegt werden, um das bestmögliche Behandlungsergebnis und Outcome zu realisieren [21].

### Verteilung von Patienten

Die Verlegung und Verteilung von bereits im Krankenhaus befindlichen Patienten oder neu Zugewiesener nach initialer Kategorisierung und Stabilisierung ist eine mögliche weitere taktische Maßnahme.

Hierbei ist zu berücksichtigen, dass Art und Dauer von Transport und Verlegung sich nicht nachteilig auf das Behandlungsergebnis auswirken sollten. Das bestmögliche erreichbare Behandlungsergebnis ist in dieser Situation abhängig von der Zielsetzung und Priorisierung der Behandlung und den lokal verfügbaren Kapazitäten und Ressourcen [8].

Eine kurzfristige Verlegung von Patienten nach initialer Stabilisierung am Schadensort oder im erstversorgenden Krankenhaus führt in den benachbarten Krankenhäusern zu einer zusätzlichen Belastung. Ein in der SARS-CoV-2-Pandemie deutschlandweit entwickeltes und erfolgreich angewandtes System zur Verteilung von Patienten bei einer Kapazitätsüberlastung ist das Kleeblattsystem. Es wurde im Rahmen des Ukraine-Krieges erweitert und wird nun auch zur Verteilung von Patienten aus der Ukraine eingesetzt. Dieses System hat sicherlich das Potenzial, auch bei den Szenarien A bis C zum Einsatz zu kommen. Aus den Erfahrungen der vergangenen Jahre müsste das Kleeblattsystem aber um eine elektronische Komponente zum geschützten Onlineaustausch patientenbezogener medizinischer Daten erweitert werden, um es als Just-in-time-Führungsmittel vollumfänglich nutzen zu können [11].

### Verteilung von Personal und Material

Auch die Zuteilung und Verbringung von Personal und Material in das erstversorgende, im Schwerpunkt betroffene Krankenhaus, kann eine Entlastung bedingen. Hierbei ist zu berücksichtigen, dass im Rahmen der Vorbereitung die organisatorischen und logistischen Grundlagen etabliert werden sollten, dass das Material auch kurzfristig genutzt und betrieben bzw. das Personal auch eingesetzt werden kann und am Patienten zum Einsatz kommt. Dies kann z. B. durch gegenseitige Kooperationsvereinbarungen erreicht werden, wie sie für Konsildienste in den Traumanetzwerken etabliert sind. Sie müssen ggf. erweitert oder angepasst werden und können eine gegenseitige Unterstützung in Ausnahmesituation gewährleisten. Auch stellt die gemeinsame Beschaffung und Bewirtschaftung von Notfallsieben und Fixateuren eine Möglichkeit der gemeinsamen Vorbereitung und Steigerung der chirurgischen Behandlungskapazität dar [15, 22].

**Figure 2:**
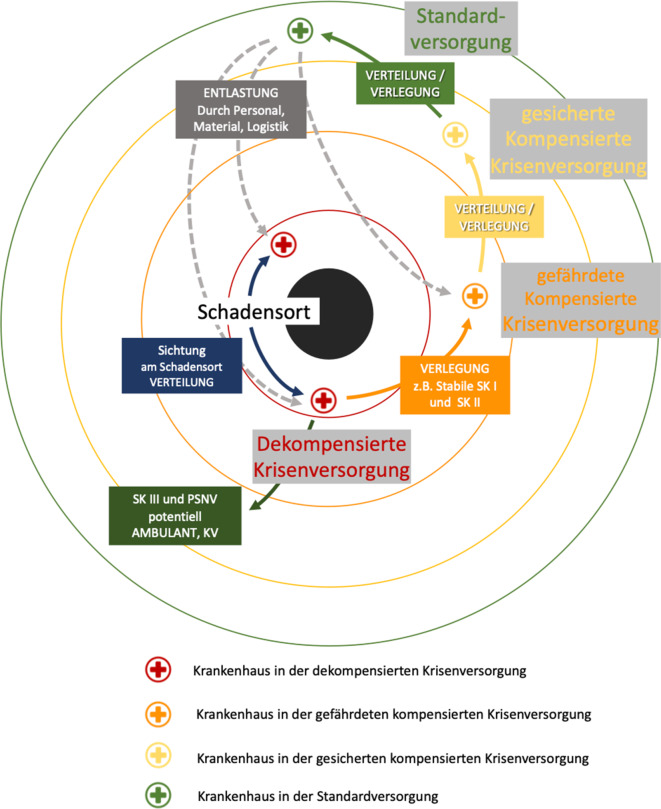

Um eine Zone der gefährdeten kompensierten Krisenversorgung schließen sich weniger oder gar nicht betroffenen Region an. Das Versorgungslevel sollte hier im Stadium der *kompensierten und gesicherten Krisenversorgung oder aber der Standardversorgung* gewährleistet sein (Abb. 2).

In Lagen, in denen längerfristig von einer regionalen dekompensierten Krisenversorgung mit begleitend gefährdeter oder gesicherter kompensierter Krisenversorgung auszugehen ist (Szenario B und C), können auch strategisch Material, Personal oder mobile Behandlungseinheiten entsandt werden. Welche kompensierenden Maßnahmen hier erforderlich sind, hängt entscheidend von dem Bedarf und den grundsätzlich vorhandenen Möglichkeiten und Kapazitäten ab und setzt eine ursachenorientierte Lagebeurteilung voraus.

### Führung und Lagedarstellung

Um eine solche Schadenslage überregional bewältigen zu können ist eine übergeordnete Führung und ein einheitliches Lagebild essenziell. Eine Möglichkeit ein solches Lagebild einfach und schnell regional und überregional zu erstellen, ist das sog. Windmühlenmodell, das in Bayern als Führungs- und Lagewerkzeug im Rahmen der SARS-CoV-2-Pandemie erfolgreich eingesetzt wurde [19].

**Figure 3:**
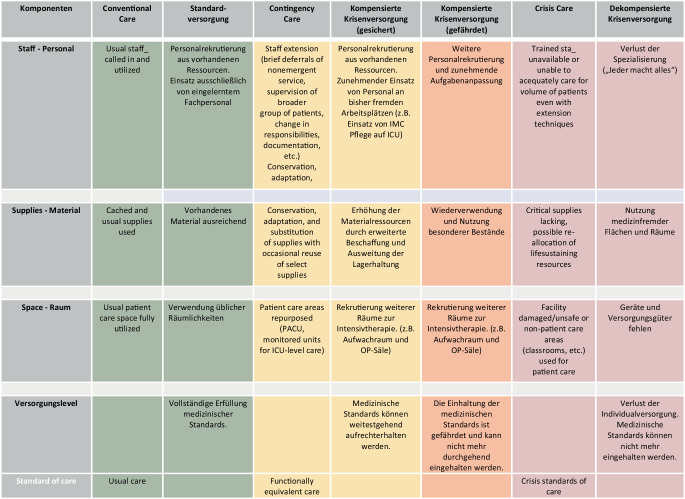

Eine Übersicht über die verschiedenen Versorgungslevel und die möglichen Kompensationsmaßnahmen in einer Mangelsituation geben die Abb. 3 und 4. Die aufgeführten Begrifflichkeiten haben sich im internationalen Sprachgebrauch etabliert [12, 19, 20].

**Figure 4:**
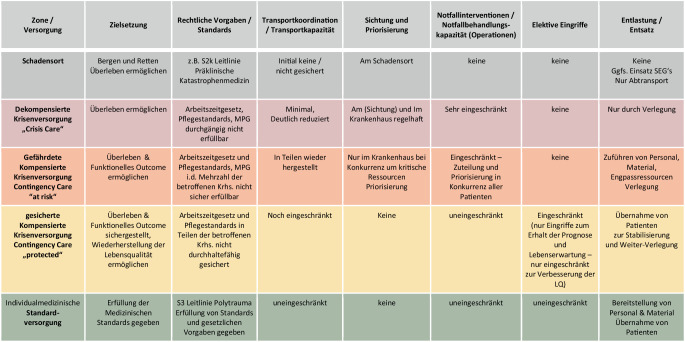

### Mittelfristige Reaktionsmöglichkeiten in Abhängigkeit von der Dauer der Szenarien

Bedingt das Szenario durch Dauer und Anzahl der Verletzten und Erkrankten eine längerfristige Belastung eines oder mehrerer Krankenhäuser einer Region, vergrößert sich unter Umständen die Zone, in der es zu einer *gesicherten kompensierten Krisenversorgung* oder *gefährdeten kompensierten Krisenversorgung* kommen kann.

Die grundsätzlichen Reaktionsmöglichkeiten sind die gleichen wie bei einem kurzzeitigen Ereignis. Allerdings kann aufgrund der Dauer auch aus weiter entfernten Ballungszentren oder Versorgungsnetzwerken Personal und Material zur lokalen Unterstützung herangeführt werden. Dies kann z. B. zwischen Krankenhäusern, die den gleichen Träger und den gleichen Versorgungslevel haben, im Vorfeld vereinbart vorbereitet werden.

Ebenfalls erscheint es sinnvoll, Patienten auch über längere Distanzen zu verlegen. Aufgrund der Dauer und der längeren Transportwege ist davon auszugehen, dass die Grenzen etablierter Versorgungsstrukturen (z. B. der lokal betroffenen Traumanetzwerke) oder auch Landesgrenzen überschritten werden.

Um hier eine homogene Verteilung von Personal, Material und Patienten zu erwirken, sind überregionale Absprachen, Konzepte und eine Verteilungskoordination erforderlich. Hier kann man das Gemeinsame Melde- und Lagezentrum des Bundes (GMLZ) oder Strukturen, wie das zur Verlegung von COVID-19(„coronavirus disease 2019“)-Patienten etablierte „Kleeblatt“ oder IVENA (Interdisziplinärer Versorgungsnachweis), qualifizieren, um eine solche Führungs- und Verteilungsstruktur aufzubauen [10, 16].

Alternativ können aus weiter entfernten (Ballungs‑)Zentren Material und Personal mobilisiert werden, um lokal zu unterstützen. Gleichzeitig müssen in weit entfernten Versorgungsnetzwerken Behandlungskapazitäten kurzfristig bereitgestellt werden, um Verletzte oder Erkrankte aufzunehmen.

## Diskussion

Übergeordnetes Ziel in jeder Schadenslage muss es sein, eine kompensierte Krisenversorgung in den möglichen Szenarien längstmöglich aufrechtzuerhalten oder frühestmöglich wieder herzustellen. Dies wurde an drei theoretischen Szenarien und den sich daraus ergebenden Belastungen für das Gesundheitssystem eingangs orientierend erläutert.

Zur Darstellung und Bewertung bedarf es einer einheitlichen Nomenklatur der unterschiedlichsten Versorgungsmöglichkeiten von der *dekompensierten Krisenversorgung* über die *gefährdete *und *ungefährdete kompensierte Krisenversorgung* bis zur *Standardversorgung* [12, 19, 20].

Die beispielhafte Abgrenzung zwischen den spezifischen Merkmalen der einzelnen abgestuften Krisenversorgung zur Bewältigung einer Schadenslage wird in dieser Arbeit dargestellt. Die Beurteilung der eigenen realistischen Lage und Reaktionsmöglichkeiten erfordert zusätzlich ein kontinuierliches Lagebild von der Situation vor Ort, im eigenen Krankenhaus, in der lokalen oder regionalen Gebietskörperschaft und darüber hinaus.

Die Werkzeuge für die Erfassung und Darstellung dieses Lagebildes sind zum Teil schon etabliert (z. B. Windmühlenmodell [19]) und basieren auf dieser einheitlichen Nomenklatur. Beim Windmühlenmodell wird das eigene Krankenhaus bezüglich der Lagebeurteilung in Funktionsbereiche eingeteilt. Jeder Flügel entspricht einem Bereich und bewertet dann eigenständig, in welchem Stadium der Krisenversorgung er sich befinden bzw. welche Standards, Regularien, Vorgaben nicht mehr gewährleistet werden können (siehe oben analog zu Abb. 3). Das Krankenhaus beurteilt dann den eigenen Status bezüglich der Krisenversorgung anhand dieses Lagebildes und meldet dies an die Gebietskörperschaft. Auf Ebenen der Gebietskörperschaft repräsentiert dann wiederum jedes Krankenhaus einen Flügel der Windmühle. So entwickelt sich ein kontinuierliches Lagebild auf jeder Ebene für die jeweils darüberliegende.

Einheitliche Kommunikationsstrukturen und bekannte Behandlungskapazitäten sind eine weitere Voraussetzung für die Bewältigung einer Schadenslage.

Die Verbreitung dieser Kenntnisse gilt es, weiter zu fördern und zusammen mit den einheitlichen Begrifflichkeiten zur Grundlage für regionale, überregionale oder nationale taktisch-strategische Entscheidungen zu machen. Ziel muss es bleiben, durch eine adäquate Vorbereitung schon primär eine kompensierte Krisenversorgung so lange wie möglich sicherzustellen. Ein umfassendes Konzept zur Aufrechterhaltung der medizinischen Versorgung wurde ganz aktuell veröffentlicht und bestätigt viele der hier genannten strategischen und taktischen Vorgehensweisen [13].

Eine taktisch-strategische Versorgung mit dem Ziel das Überleben möglichst vieler zu ermöglichen, ist hierbei nur der erste Schritt. Zusammen mit den dargestellten Kompensationsmechanismen (z. B. Verlegung von Patienten, Zufuhr und Mobilisation von Ressourcen) zur frühestmöglichen Begrenzung der akuten Mangelsituation und zum Wiedererreichen mindestens der kompensierten Krisenversorgung dient alles dem gemeinsamen übergeordneten Ziel, die bestmögliche Versorgung für möglichst alle Patienten zu realisieren [4].

Es darf aus den Ausführungen keinesfalls gefolgert werden, die Vorbereitung a priori auf Kosten der Standards zu limitieren. Im Gegenteil, die hier gezeigten Konzepte legen nahe, dass es bezüglich der Vorbereitung auf Großschadenereignisse und Katastrophen noch erheblichen Bedarf gibt! Zum Beispiel braucht es Festlegungen, auf welche Szenarien eine Vorbereitung erfolgen soll und in welchem Umfang. Darüber hinaus ist fortlaufend zu prüfen, ob sich nicht durch die aktuelle (politische) Lage neue Herausforderungen ergeben. Diese sind offen zu kommunizieren, um allen Beteiligten (insbesondere den betroffenen Krankenhäusern) die Gelegenheit zu geben, ihre Planungen, Krankenhausalarm- und -einsatzpläne (KAEP) und Vorbereitungen dahingehend anzupassen.

Zusätzlich sind – vergleichbar der qualitativen Verbesserung der individualmedizinischen Schwerstverletztenversorgung durch die Anwendung der entsprechenden S3-Leitlinie, die Einführung standardisierter Kursformate (z. B. ATLS-Kurse), des Traumaregisters, der Traumanetzwerke und die Umsetzung der Vorgaben des Weissbuches der Schwerstverletztenversorgung der DGU – die hierfür etablierten Strukturen durch einheitliche bundesweite Konzepte und Begrifflichkeiten in der medizinischen Krisenversorgung einer Katastrophe oder einer Großschadenslage weiterzuentwickeln und einzubinden [14].

Da hier vor allem der chirurgische und notfallmedizinische Behandlungsbedarf bei den erwartbaren Katastrophen- und Großschadenslagen im Vordergrund stehen wird, ist der politische und fachliche Konsens zu fördern, dass die Finanzierung von Großübungen und die flächendeckende Etablierung von Kurskonzepten (TDSC, ACT [Acute Care in Trauma]) usw. zur Vermittlung notfallchirurgischer Fertigkeiten (z. B. Versorgung von Verletzungen der Körperhöhlen) und taktisch-strategischer Konzepte zur Bewältigung einer Schadenslage durch Bund und Länder gesichert ist.

Weiterhin gilt es, einen allgemeinen Konsens über Begrifflichkeiten, Art der Versorgung und die taktisch strategischen Grundsätze zur Bewältigung einer Katastrophe oder Schadenslage weiter zu etablieren.

In dieser Zielsetzung erfolgt aktuell auch die Ausarbeitung der ist S2k-Leitlinie „Klinische Katastrophenmedizin für Deutschland“ (LeiKliKatMeD) gemeinsam mit allen anderen beteiligten Fachgesellschaften.

Die Verantwortlichkeit für die fachliche und strukturelle Umsetzung zur Optimierung einer flächendeckenden notfall- und katastrophenmedizinischen Versorgung und ihrer Vorbereitung liegt aber je nach angenommenem Szenario und Schadenslage bei Bund, Ländern und/oder Gebietskörperschaften. Hier wird der aktuell in Entwicklung befindlichen Gesetzgebung für die Strukturreform der Krankenhäuser, die Sicherstellung und Neustrukturierung der stationären Notfallversorgung und der Weiterentwicklung der Krankenhausfinanzierung eine große Bedeutung zukommen. Es ist hier nicht immer für alle Beteiligten klar ersichtlich, dass auch die flächendeckende katastrophenmedizinische und notfallchirurgische Versorgung der Bevölkerung vollumfänglich in die Konzepte und Überlegungen miteinbezogen wird. Bei der angestrebten Weiterentwicklung und stetigen Verbesserung ist zu diskutieren, ob eine gemeinsame einheitliche Führungsstruktur und Institution von Bund und Ländern für die Konzeption, Etablierung und Führung des Schutzes der Bevölkerung in einer solchen Ausnahmesituation verantwortlich sein sollte.

Bezogen auf die aktuell vorhandenen Versorgungskapazitäten und Traumanetzwerke ist es die Herausforderung, die Entwicklung unseres Gesundheitssystems auch unter dem Aspekt der allgemeinen Traumaversorgung, der katastrophenmedizinischen Krisenversorgung zu gestalten. Aus Sicht der medizinischen Fachgesellschaften (z. B. DGU und Deutsche Arbeitsgemeinschaft Krankenhaus-Einsatzplanung [DAKEP]) können hierbei Lösungen, Konzepte und Zertifizierungen angeboten werden, die dann inhaltlich vorgeben, wie sich Kliniken in und für Schadenslagen vorbereiten können.

## Schlussfolgerung

Im Rahmen einer Großschadenslage oder Katastrophe muss es das Ziel sein, in allen Bereichen das Stadium einer *kompensierten Krisenversorgung*, insbesondere der chirurgischen Versorgung der Verletzten, zu erhalten oder wieder herzustellen. Für die Lagebeurteilung und Kommunikation sind einheitliche Begrifflichkeiten und ein gemeinsames Verständnis erforderlich, welche die vorliegende Arbeit darstellt.

Gleichzeitig wird verdeutlicht, dass je nach Ausdehnung der Schadenslage die kompensierte Krisenversorgung nur aufrechterhalten werden kann, wenn unabhängig von den Landesgrenzen Ressourcen mobilisiert werden. Hierfür ist eine übergeordnete zentrale Führungsstruktur erforderlich und Schwellendefinitionen für Ereignisse, Aufgaben und Kompetenzen einer solchen Führungsstruktur sind stetig anzupassen oder zu etablieren, um handlungsfähig zu sein.

Für die Bereitstellung chirurgischer und notfallmedizinischer Behandlungskapazitäten zur Bewältigung einer (Groß‑)Schadenslage oder Katastrophe ist die Darstellung eines kontinuierlichen Lagebildes mit aktuellen Ressourcen und Strukturdaten der Krankenhäuser in der betroffenen Region erforderlich. Grundsätzlich können zur Bewältigung Patienten, Material und Personal verschoben werden. Dies erfordert aber Informationen zur Verfügbarkeit von Ressourcen. Aktuell gibt es zwar etablierte Lösungen zur Darstellung von Behandlungskapazitäten, aber z. B. keine flächendeckenden, belastbaren online verfügbaren Daten zur detaillierten Ausstattung, zu Räumlichkeiten und Ausweichliegenschaften im Sinne eines aktuellen Krankenhauskatasters. Eine Abschätzung der verfügbaren chirurgischen Behandlungskapazität liefert eine Onlinebefragung der in den Traumanetzwerken der DGU zertifizierten Krankenhäuser in dieser Ausgabe von *Die Chirugie* [9].

Hierbei kann ein Massenanfall von Verletzten oder Erkrankten initial lokal und vorübergehend aufgrund der Dynamik zu einer *dekompensierte Krisenversorgung*. Um dies möglichst im Vorfeld zu vermeiden, gilt es, die Krankenhäuser und Gebietskörperschaften bei ihren Bemühungen zu unterstützen, die erreichten Qualitätsstandards der Traumaversorgung in Krisensituationen aufrechtzuerhalten und die Vorbereitung auf solche Ereignisse zu intensivieren. Nur so lässt sich das Ziel der möglichst langen Aufrechterhaltung einer kompensierten Krisenversorgung oder einer schnellstmöglichen Rekompensation erreichen.

Insgesamt müssen die Fähigkeiten zur medizinischen Versorgung in Katastrophen und Großschadenslagen derart optimiert werden, dass eine realistische Möglichkeit zur Gewährleistung einer kompensierten Krisenversorgung besteht.
